# Associations between employees’ alcohol consumption, insomnia and HR management strength

**DOI:** 10.1093/occmed/kqae100

**Published:** 2024-11-09

**Authors:** T-H Dao-Tran, K Townsend, R Loudoun, A Wilkinson, C Seib

**Affiliations:** Centre for Health Services Research, Faculty of Medicine, University of Queensland, Brisbane, QLD 4006, Australia; Centre for Work, Organisation, and Well-being, Griffith University, Brisbane, QLD 4111, Australia; Centre for Work, Organisation, and Well-being, Griffith University, Brisbane, QLD 4111, Australia; Centre for Work, Organisation, and Well-being, Griffith University, Brisbane, QLD 4111, Australia; School of Nursing and Midwifery, Griffith University, Logan, QLD 4131, Australia

## Abstract

**Background:**

Understanding of hazardous alcohol drinking and insomnia among Australian ambulance personnel is limited. Australian ambulance organizations have strengthened their organizational human resource management (HRM) to promote their employees’ healthy lifestyles, health and well-being.

**Aims:**

To describe the prevalence of hazardous alcohol consumption and insomnia among Australian ambulance personnel and to explore their associations with the organizational HRM strength.

**Methods:**

This cross-sectional study was conducted on 492 ambulance personnel randomly selected from three Australian states. The Alcohol Use Disorders tool, The Insomnia Severity Index and the Perceived HRM System Strength instrument measured alcohol consumption, insomnia and HRM strength. Descriptive analyses, bivariate association analyses and general linear models were used for data analysis.

**Results:**

Twenty per cent of Australian ambulance personnel consumed alcohol at a hazardous level and 68% experienced clinically significant insomnia. There was no significant association between organizational HRM strength and ambulance personnel’s hazardous alcohol consumption. There was a significant association between organizational HRM strength (consensus) and ambulance personnel’s insomnia experience.

**Conclusions:**

Hazardous alcohol consumption and insomnia were concerns among Australian ambulance personnel. Even though strengthening the HRM system might reduce their experience of insomnia, simply strengthening the HRM system could not reduce their hazardous alcohol consumption.

## Teaser

Australian ambulance personnel consumed alcohol at a hazardous level (20%) and experienced insomnia (68%). The organizational human resource management system strength was associated with their reduced experience of insomnia but not associated with reduced alcohol consumption. Multiple strategies should be implemented to manage their hazardous alcohol consumption. Information on their support access should be kept strictly confidential to promote their utilization of the provided support.

## Introduction

Ambulance personnel are often exposed to traumatic events and do shift work as the nature of work [1,2]. Drinking alcohol is one of their coping strategies for occupational stress [3]. On the other hand, shift work can increase their risk of insomnia [4]. Research has shown that ambulance personnel often have issues with alcohol consumption and insomnia [3,5]. Since hazardous alcohol consumption and insomnia can lead to multiple negative influences on their work performance, health and well-being [6–8], research into strategies to manage their hazardous alcohol consumption and insomnia is important.

Existing evidence of Australian ambulance personnel suggests that 15% consume more than 10 standard drinks weekly [9], and 20–80% have sleep problems [4,10]. However, the studies conducted in Australia often have small sample sizes (*n* = 48–60 [9,11] and *n* = 136 [4]). While Australian ambulance personnel include paramedics [12] and non-paramedic staff (e.g. patient transport officers, emergency medical dispatchers, call takers) [13], the previous study was conducted on paramedics only but did not include non-paramedic staff [4]. One study measures sleep problems using a single item [9]. These raise methodological concerns and limit the generalization of the findings to a broader Australian ambulance workforce. Thus, more research into hazardous alcohol consumption and insomnia among Australian ambulance personnel is warranted.

To support ambulance personnel in managing their lifestyles, health and well-being, ambulance organizations have provided different support services [14]. However, ambulance personnel have not sought internal support because of mental health-related stigma [15] or hold concerns about confidentiality and the potential negative impact on their career [16]. Apart from pre-existing support, large Australian ambulance organizations have also strengthened their human resource management (HRM) systems with an expectation that it will promote their employees’ healthy lifestyles, health and well-being, including hazardous alcohol consumption and insomnia, fatigue, post-traumatic stress disorders, anxiety, stress and depression [17]. Approaches applied to strengthen their HRM include having realistic job previews, sophisticated hiring practices, extensive employee orientation and induction programmes, the introduction of frequent, thorough and critical performance management programmes, increased training opportunities and expectations, focussed internal promotions where possible with career planning opportunities, improved processes for employee voice; decentralized problem-solving teams, increased average pay and increased the number of staff to allow flexibility in a crisis [18].

Despite the decades-long search, HRM theory development remains problematic [19]. It is unclear if strengthening the HRM system can reduce employees’ alcohol consumption and insomnia. This study aims to describe the prevalence of hazardous alcohol consumption and insomnia among Australian ambulance personnel and examine their associations with HRM strength. Based on current literature, it is hypothesized that HRM strength is significantly associated with reduced hazardous alcohol consumption and insomnia [20].

## Methods

This cross-sectional study is part of a more extensive survey conducted in three Australian states in June and July 2017. Ethical approval was obtained from the Ethics Committee of Griffith University (No: 2016/702). Ethical approval required not disclosing the name of the participating states. The Unions were collaborative partners in this research project. Research and development approvals from participating organizations were not required.

Call centre staff who were not ambulance personnel nor Union members employed by the Unions used the Union member lists (90% of ambulance personnel workforce, *n* = 4959) and randomly contacted the Union members to invite their participation in the research project. The recruitment was conducted until the number of participants met 10% of the ambulance personnel in each state. The Union did not have non-union contacts; therefore, they could not invite them to participate. Administrative staff and staff on leave were excluded from participating in the study. Participants were informed that their participation was voluntary and that their decision not to participate would not have a negative impact on their relationship with the employer or the Unions.

In total, Call centre staff invited 1216 ambulance personnel who satisfied the criteria: 724 chose not to participate and 492 ambulance personnel agreed to participate, a response rate of 41%. This sample size (*n* = 492) satisfied the required sample size for this study because the minimum sample size for statistical data analysis power was 150, given that 10 participants were needed for one variable in the multiple regression analysis [21].

Literature suggested that online surveys had a higher response rate [22], and phone calls could further increase the response rate [23] than traditional mail. In the co-design process with the research partners, the Unions also supported telephone-administered online interviews because they believed it would allow data collection to be completed within the agreed timeframe with a higher response rate.

The research team developed the Qualtrics online survey platform, accessible through a public link, and trained call centre staff to interview the participants and collect data over the phone. Prior to conducting the interviews, consent was obtained from all participants. If the survey was not completed in a single call, mutually agreeable call-back times were arranged until the survey was complete.

Where possible, validated scales were included in the survey to measure variables of interest. The Alcohol Use Disorders (AUDIT) tool [24] was used to assess participants’ alcohol consumption. This self-reported scale has 10 items, asking participants to indicate their use of alcoholic beverages during the past 12 months. The responses are scored on a 5-point rating scale (from 0 to 4), with options 1 and 3 excluded from questions 9 and 10, then summed to obtain a total score between 0 and 40. A cut point of 8 indicates that a person has hazardous alcohol consumption. This validated scale had a Cronbach’s alpha of 0.85 [25].

The Insomnia Severity Index [26] examined participants’ experience of insomnia. This scale has seven items, asking participants to rate their experience with sleep in the last 2 weeks. The responses are rated on a 5-point scale (0–4). The total score ranges from 0 to 28. Scores are categorized as no clinically significant insomnia (0–7), sub-threshold insomnia (8–14), moderate insomnia (15–21) and severe insomnia (22–28). This scale has been validated and has Cronbach’s alphas of 0.76–0.78 [26].

Participants were also asked to answer the Perceived HRM System Strength instrument [27]. This instrument has 31 self-report items, asking participants to rate how much they agree with each statement about their organization. The response options are strongly disagree (1), somewhat disagree (2), neither agree nor disagree (3), agree (4) and strongly agree (5). The instrument has three major subscales, including distinctiveness (10 items), consistency (9 items) and consensus (12 items). Higher total scores indicate a stronger HRM system strength. This scale has been validated and has a Cronbach’s alpha of 0.90 [27].

Covariates: age, sex, education (high school, diploma, bachelor’s degree, post-graduate), marital status (married/living in a partnership or not), number of dependents (zero, one, two, three. or more), job tenure (in years), working role (paramedics or not), working location (metropolitan city, regional city/town, rural areas), the average actual number of working hours per week including overtime and number of nightshifts worked per month [28,29].

SPSS 29.0 was used to analyse the data [30]. Descriptive analyses (frequency, percentage, median, interquartile [IQR] range). Because continuous variables (except for HRM strength, distinctiveness, consensus and consistency) were not normally distributed, non-parametric tests were used. Chi-square and Mann–Whitney tests were used to describe the difference between those with and without hazardous alcohol consumption and between those with and without insomnia.

Two general linear models (GLMs) were used to investigate the associations between HRM strength, and alcohol consumption and insomnia. Mann–Whitney, Kruskal–Wallis and Spearman Rho tests were used to preliminarily examine the bivariate associations between personal and work-related characteristics, HRM strengths (distinctiveness, consistency and consensus) and alcohol consumption, insomnia as appropriate. The GLM included only variables significantly associated with alcohol consumption and insomnia in the bivariate association analyses. Model 1 examined the association between HRM strength and alcohol consumption while controlling the effect of personal and work-related characteristics and insomnia. Model 2 examined the associations between HRM system strength and insomnia while controlling the effect of personal, work-related characteristics and alcohol consumption. A statistical significance level for the bivariate correlation tests and generalized linear model analyses was set at 0.05.

## Results

All participants (*n* = 492, 100%) completed the survey. Their median age was 45 years (IQR = 35.0–52.0). Most participants were paramedics (84%). Approximately two-thirds were males (68%), married or had a partner (78%), and worked in Metropolitan cities (61%). More than 40% completed a bachelor’s degree (44%) or had no dependents (44%). On average, their median job tenure was 14 years (IQR = 8.0–24.0). They worked 48 hours/week (IQR = 42.0–51.5), including six nights per month (IQR = 0.0–8.0). Around 20% of participants (*n* = 98) reported hazardous alcohol consumption (AUDIT score ≥8). The prevalence of clinically significant insomnia (Insomnia Severity Index score ≥8) was 68% (*n* = 335), including 48% with sub-threshold insomnia (*n* = 236) and about 20% with moderate insomnia (*n* = 99). The mean HRM strength score was 77.70 (standard deviation [SD] = 17.57) out of a possible score of 155. The mean scores for distinctiveness were 23.70 out of 50 (SD = 6.86), consistency was 22.30 out of 45 (SD = 6.21), and consensus was 31.71 out of 60 (SD = 6.80).

Males were more likely to consume alcohol at hazardous levels than females (22% vs 15%, *P* < 0.05). Those with clinically significant insomnia were more likely to drink alcohol at hazardous levels than those without (24% vs. 12%, *P* < 0.001). No significant differences existed between those with and without hazardous alcohol consumption regarding age, education, marital status, number of dependents, working location, working roles, job tenure, working hours or number of night shifts worked (see Table 1).

**Table 1.** Factors associated with hazardous alcohol consumption (*n* = 492)

		With hazardous alcohol consumption (*n* = 98, 20%) *n* (%)	Without hazardous alcohol consumption (*n* = 394, 80%) *n* (%)
Age (Median [IQR])		45.00 [35.00–52.00]	45.00 [34.50–52.00]
Sex	Male	75 (22)	260 (78)^*^
	Female	23 (15)	134 (85)
Education	High school	7 (20)	29 (80)
	Diploma	29 (18)	136 (82)
	Bachelor	46 (21)	171 (79)
	Post-graduate	16 (22)	58 (78)
Marital status	Married/ Partner	23 (21)	87 (79)
	Others	75 (20)	307 (80)
Number of dependents	0	45 (21)	173 (79)
	1	18 (21)	67 (79)
	2	23 (19)	99 (81)
	3+	12 (18)	55 (82)
Working location	Metropolitan	60 (20)	241 (80)
	Regional area	25 (22)	87 (78)
	Rural area	13 (17)	66 (84)
Working roles	Non-paramedic	12 (16)	65 (84)
	Paramedic	86 (21)	329 (79)
Job tenure (years) (Median [IQR])		14.0 [8.38–24.00]	14.0 [8.00–24.25]
Actual working hours/week (Median [IQR])		46.0 [43.00–52.50]	48.0 [42.00–51.00]
Number of night shifts worked (Median [IQR])		6.0 [0–8.00]	6.0 [0–8.00]
Insomnia	No	139 (89)	18 (12)^***^
	Yes	255 (76)	80 (24)

*n*, number; %, valid percentage; IQR, interquartile range.

^*^ *P* < 0.05,

^***^ *P* < 0.001.

Paramedics were more likely to experience clinically significant insomnia than non-paramedic staff (70% versus 56%, *P* < 0.05). Those with hazardous alcohol consumption were more likely to experience insomnia than those without hazardous alcohol consumption (82% versus 65%, *P* < 0.001). No significant differences existed between those with and without clinically significant insomnia regarding age, sex, education, marital status, number of dependents, working location, job tenure, working hours, or number of night shifts worked (see Table 2).

**Table 2.** Factors associated with clinically significant insomnia (*n* = 492)

		With insomnia (*n* = 335, 68%) *n* (%)	Without insomnia (*n* = 157, 22%) *n* (%)
Age (Median [IQR])		45.0 [34.75–52.00]	45.0 [35.00–53.50]
Sex	Male	224 (67)	111 (33)
	Female	111 (71)	46 (29)
Education	High school	21 (58)	15 (42)
	Diploma	108 (66)	57 (35)
	Bachelor	155 (71)	62 (29)
	Post-graduate	51 (69)	23 (31)
Marital status	Married/ Partner	255 (67)	127 (33)
	Others	80 (73)	30 (27)
Number of dependents	0	140 (64)	78 (36)
	1	56 (66)	29 (34)
	2	92 (75)	30 (25)
	3+	47 (70)	20 (30)
Working location	Metropolitan	210 (70)	91 (30)
	Regional area	72 (64)	40 (36)
	Rural area	53 (67)	26 (33)
Working roles	Paramedic	292 (70)	123 (30)^*^
	Non-paramedic	43 (56)	34 (44)
Job tenure (years) (Median [IQR])		13.0 [8.00–23.00]	15.0 [8.75-26.00]
Actual working hours/week (Median [IQR])		48.0 [42.25–51.50]	48.0 [42–51.75]
Number of night shifts worked (Median [IQR])		6.0 [0–8.00]	5.0 [0–8.00]
Hazardous alcohol consumption	No	255 (65)	139 (35)^***^
	Yes	80 (82)	18 (18)

*n*, number; %, valid percentage; IQR, interquartile range.

^*^ *P *< 0.05,

^***^ *P* < 0.001.

Spearman rho tests suggested that distinctiveness was negatively correlated with alcohol consumption (*µ* = −0.108, *P* < 0.05) and experience of insomnia (*µ* = −0.154, *P* < 0.001). Consistency was negatively correlated with alcohol consumption (*µ* = −0.134, *P* < 0.01) and experience of insomnia (*µ* = −0.126, *P* < 0.01). Consensus was negatively correlated with the experience of insomnia (*µ* = −0.207, *P* < 0.001).

Model 1 indicates that after controlling for the potential effects of personal, work-related characteristics and insomnia, HRM strength (including distinctiveness, consistency and consensus) did not significantly impact alcohol consumption (see Table 3).

**Table 3.** Associations between HRM strength and alcohol consumption (*n* = 492)

Model 1		*β*	95% CI
Intercept		4.612	0.836 to 8.389^*^
Sex	Male	0.970	0.156 to 1.783^*^
	Female	Ref	Ref
Education	High school	−0.459	−1.970 to 1.052
	Diploma	−0.229	−1.803 to 1.344
	Bachelor	−0.677	−2.384 to 1.030
	Post-graduate	Ref	Ref
Married status	No	−0.058	−0.994 to 0.878
	Yes	Ref	Ref
Number of dependents	0	0.729	−0.412 to 1.870
	1	0.488	−0.825 to 1.801
	2	0.676	−0.535 to 1.886
	3+	Ref	Ref
Age		0.027	−0.026 to 0.080
Paramedic status	No	−0.634	−1.753 to 0.485
	Yes	Ref	Ref
Work location	Metropolitan	0.452	−0.605 to 1.508
	Regional	0.498	−0.693 to 1.689
	Rural	Ref	Ref
Job tenure		−0.049	−0.106 to 0.008
Working hours		−0.023	−0.053 to 0.007
Number of nightshifts		0.035	−0.055 to 0.125
Insomnia		0.081	−0.002 to 0.164
Distinctiveness		−0.044	−0.128 to 0.040
Consistency		−0.077	−0.173 to 0.019
Consensus		0.045	−0.028 to 0.117

*β*, coefficient; CI, confident interval. Sex, marital status, education, number of dependents, paramedic status and work location are categorical variables. Age, job tenure, working hours, number of nightshifts, insomnia experience, distinctiveness, consistency, consensus and alcohol consumption are continuous variables.

^*^ *P* < 0.05.

Model 2 indicates that after controlling for the potential effects of personal and work-related characteristics and alcohol consumption, consensus significantly impacted insomnia (*β* = −0.134, *P* < 0.001) (see Table 4).

**Table 4.** Associations between HRM strength and insomnia (*n* = 492)

Model 2		*β*	95% CI
Intercept		12.621	8.591 to 16.651^***^
Sex	Male	−0.691	−1.591 to 0.210
	Female	Ref	Ref
Education	High school	0.447	−1.220 to 2.114
	Diploma	0.738	−0.997 to 2.473
	Bachelor	0.054	−1.830 to 1.939
	Post-graduate	Ref	Ref
Married status	No	0.892	−0.137 to 1.922
	Yes	Ref	Ref
Dependents	0	−0.742	−2.001 to 0.518
	1	−0.273	−1.722 to 1.176
	2	0.690	−0.645 to 2.026
	3+	Ref	Ref
Age		0.023	−0.035 to 0.081
Paramedic	No	−1.585	−2.813 to −0.357^*^
	Yes	Ref	Ref
Work location	Metropolitan	0.529	−0.636 to 1.695
	Regional	0.170	−1.145 to 1.485
	Rural	Ref	Ref
Job tenure		−0.008	−0.071 to 0.055
Working hours		0.006	−0.027 to 0.039
Number of nightshifts		0.095	−0.004 to 0.194
Hazardous alcohol consumption		0.098	−0.003 to 0.199
Distinctiveness		−0.027	−0.120 to 0.066
Consistency		0.015	−0.091 to 0.121
Consensus		−0.134	−0.213 to −0.055^***^

*β*, coefficient; CI, confident interval. Sex, marital status, education, number of dependents, paramedic status and work location are categorical variables. Age, job tenure, working hours, number of nightshifts, alcohol consumption, distinctiveness, consistency, consensus and insomnia experience are continuous variables.

^*^ *P* < 0.05,

^***^ *P* < 0.001.

## Discussion

This study found that 20% of Australian ambulance personnel consumed alcohol at a hazardous level and 68% experienced clinically significant insomnia. HRM system strength was not significantly associated with their alcohol consumption; a stronger consensus of HRM was significantly associated with less insomnia. This study was an initial investigation into the associations between the organizational HRM strength and employees’ alcohol consumption and experience of insomnia. Participants were stratified and randomly selected from multiple Australian states, increasing the generalizability of results for the workforce from participating states. The study used valid and reliable instruments for data collection, improving the quality of research findings and the comparability of the findings across different populations.

However, since the study used self-report questionnaires for data collection, the study’s findings might contain recall, reporting and social desirability biases [31]. We used call centre staff who were not ambulance personnel or union members and were unlikely to know the participants to collect data for more accurate replies. This study was cross-sectional, so the causal associations could not be determined [32]. The study also collected data from three Australian states. Therefore, research in other states would assist with creating a more complete picture of Australian ambulance personnel. Although the current study had a higher response rate than previous studies conducted in the ambulance profession (41% versus 16% [33]), the current response rate still potentially produces bias if there is a difference between responders and non-responders. However, using GLMs to control the potential impact of multiple confounders for data analysis helped to manage this bias. Shift-work patterns, such as varying shift types [34], may confound the association between HRM and insomnia. However, we could not collect meaningful data on shift-work patterns among the participants as they varied considerably across organizations in the participating states. Also, data collection was conducted before the coronavirus disease 2019 (COVID-19) pandemic; alcohol consumption and insomnia among ambulance personnel may have changed after the COVID-19 pandemic. Further research is needed to determine if differences occur.

The prevalence of hazardous drinking among ambulance personnel in this study is in line with that of other studies among ambulance personnel, higher than that among other healthcare professionals and lower than among police and firefighters [35]. The prevalence of hazardous drinking among ambulance personnel is much lower than that among the general adult population in Queensland, Australia (20% versus 43%). Occupational stress may have contributed to the increase in their hazardous drinking [36]. However, ambulance personnel may have a better awareness of the consequences of hazardous drinking than the general public, which may have helped them manage their alcohol drinking.

In line with the previous study, the current study found a significant association between insomnia and alcohol drinking [37]. It is unclear if ambulance personnel with insomnia might have adopted drinking alcohol as a sleep aid to deal with their insomnia or if alcohol consumption increased the experience of insomnia. In case ambulance personnel use drinking alcohol as a sleep aid to deal with their insomnia, they need to be educated that alcohol may have initial sleep-promoting effects; the use of alcohol can also disrupt standard sleep patterns [37]. Therefore, the use of alcohol should not be considered as a strategy for insomnia management in the long term.

While theory suggested that a strong HRM system can promote employees’ healthy lifestyles, we did not find a significant association between HRM strength (distinctiveness, consistency and consensus) and alcohol consumption. For safety reasons, it is expected that ambulance personnel do not drink during working hours, and organizations should have alcohol workplace policies [38] and communicate this expectation clearly to their staff. There are two potential explanations for not finding an association between HRM strength (distinctiveness, consistency and consensus) and alcohol consumption, including the study measurement and the nature of alcohol consumption behaviour. First, even though we measured alcohol drinking, our measurement did not classify alcohol drinking occurred in and out of working hours. HRM strength (distinctiveness, consistency and consensus) might have impacted alcohol consumption at work. However, it may not have impacted employees’ alcohol consumption outside of working hours. Second, because alcohol consumption can be an addictive behaviour, multiple approaches such as motivational interviewing, cognitive behaviour therapy combined with pharmacotherapy and residential rehabilitation rather than solely strengthening the HRM system can manage this behaviour successfully [39]. Third, hazardous alcohol consumption among ambulance personnel may be related to occupational stress [36]. A psychological approach should be implemented to manage these cases [39].

The prevalence of clinically significant insomnia among Australian ambulance personnel was similar to it among other shift workers [29] but higher than it among the general adult population in Australia (68% versus 33–45%) [40]. Since insomnia can have negative influences on health and well-being, interventions to manage the experience of insomnia for Australian ambulance personnel are warranted. Since paramedics were more likely to experience insomnia than non-paramedic staff, the management of insomnia should be prioritized for paramedics. Research has found that most paramedics lack sleep hygiene knowledge and exhibit poor sleep hygiene engagement; interventions may focus on sleep hygiene to improve their insomnia [34].

The study found that a higher score in the consensus component of HRM was significantly associated with a reduced experience of insomnia among Australian ambulance personnel. Consensus refers to agreement and fairness among principal human resource decision-makers [27]. Agreement among principal human resource decision-makers refers to a shared agreement between the senior and HRM teams—but more than this, through every level of management within the organization [19,28]. This finding suggests that when the senior management team and the HRM discuss their expectations and involve all staff in a consensus agreement regarding employees’ workplace practices [20], such as error management policies [18], and ensure resources, such as staffing [18], are fairly allocated to implement the agreed practices [20], the employees will be less likely to experience insomnia. The mechanisms at play here are likely to be that when the organization has a strong level of consensus between all layers of management, providing the employees with the comfort and confidence that the organization is united in its approach, ambulance personnel will be able to finish their working day with reduced levels of stress, reducing their experience of insomnia.

In summary, findings from this study contribute to refining and enhancing the literature about Australian ambulance personnel’s alcohol consumption and experience of insomnia and provide more evidence of the associations between HRM strength and employees’ alcohol consumption and experience of insomnia.

**Key learning points**

**What is already known about this subject:**

- Alcohol drinking and insomnia are concerns among ambulance personnel.
- Australian ambulance organizations have strengthened their organizational human resource management systems to promote healthy lifestyles, health and well-being for their employees.

**What this study adds:**

- Twenty per cent of Australian ambulance personnel consumed alcohol at a hazardous level; 68% of Australian ambulance personnel experienced clinically significant insomnia.
- There was no statistically significant association between human resource management strength and ambulance personnel’s hazardous alcohol consumption.
- There was a statistically significant association between human resource management strength (consensus) and ambulance personnel’s insomnia experience.

**What impact this may have on practice or policy:**

- Strengthening H human resource management systems solely may not be able to manage ambulance personnel’ alcohol consumption effectively; multiple strategies are recommended to manage their hazardous alcohol consumption more effectively.
- Involving all employees in decision-making on expected work practices and allocating resources fairly to implement agreed-upon practices may reduce employees’ insomnia experience.

## Data Availability

This study’s data are available upon request from the corresponding author. Due to ethical restrictions, the data are not available to the public.
